# Bis(2,2′-bipyridine-κ^2^ *N*,*N*′)dichlorido­platinum(IV) dichloride monohydrate

**DOI:** 10.1107/S1600536809000725

**Published:** 2009-01-14

**Authors:** Nam-Ho Kim, In-Chul Hwang, Kwang Ha

**Affiliations:** a School of Applied Chemical Engineering, The Research Institute of Catalysis, Chonnam National University, Gwangju 500-757, Republic of Korea; b Department of Chemistry, Pohang University of Science and Technology, Pohang 790-784, Republic of Korea

## Abstract

In the title complex, [PtCl_2_(C_10_H_8_N_2_)_2_]Cl_2_·H_2_O, the Pt^4+^ ion is six-coordinated in a distorted octa­hedral environment by four N atoms from the two 2,2′-bipyridine ligands and two Cl atoms. As a result of the different *trans* influences of the N and Cl atoms, the Pt—N bonds *trans* to the Cl atom are slightly longer than those *trans* to the N atom. The compound displays inter­molecular hydrogen bonding between the water mol­ecule and the Cl anions. There are inter­molecular π–π inter­actions between adjacent pyridine rings, with a centroid–centroid distance of 3.962 Å.

## Related literature

For related literature, see: Hambley (1986 ▶); Hojjat Kashani *et al.* (2008 ▶).
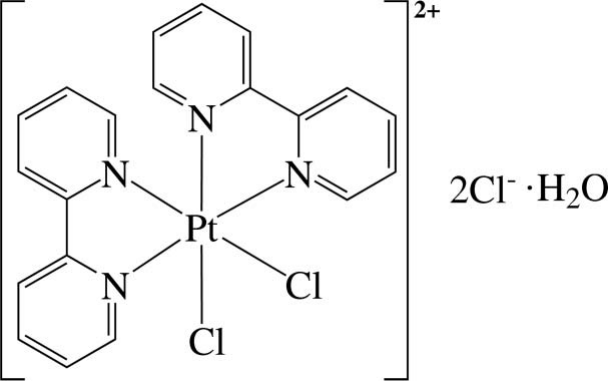


## Experimental

### 

#### Crystal data

[PtCl_2_(C_10_H_8_N_2_)_2_]Cl_2_·H_2_O
*M* *_r_* = 667.27
Orthorhombic, 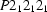

*a* = 11.1345 (12) Å
*b* = 11.5867 (12) Å
*c* = 17.0873 (19) Å
*V* = 2204.5 (4) Å^3^
*Z* = 4
Mo *K*α radiation
μ = 6.87 mm^−1^
*T* = 293 (2) K
0.35 × 0.20 × 0.15 mm

#### Data collection

Bruker SMART 1000 CCD diffractometer
Absorption correction: multi-scan (*SADABS*; Bruker, 2000 ▶) *T*_min_ = 0.251, *T*_max_ = 0.357
12649 measured reflections
4462 independent reflections
4284 reflections with *I* > 2σ(*I*)
*R*_int_ = 0.017

#### Refinement

*R*[*F* ^2^ > 2σ(*F* ^2^)] = 0.016
*wR*(*F* ^2^) = 0.038
*S* = 0.84
4462 reflections
271 parameters
H-atom parameters constrained
Δρ_max_ = 0.95 e Å^−3^
Δρ_min_ = −0.53 e Å^−3^
Absolute structure: Flack (1983 ▶), 1901 Friedel pairs
Flack parameter: −0.006 (4)

### 

Data collection: *SMART* (Bruker, 2000 ▶); cell refinement: *SAINT* (Bruker, 2000 ▶); data reduction: *SAINT*; program(s) used to solve structure: *SHELXS97* (Sheldrick, 2008 ▶); program(s) used to refine structure: *SHELXL97* (Sheldrick, 2008 ▶); molecular graphics: *ORTEP-3* (Farrugia, 1997 ▶); software used to prepare material for publication: *SHELXL97*.

## Supplementary Material

Crystal structure: contains datablocks global, I. DOI: 10.1107/S1600536809000725/bt2846sup1.cif

Structure factors: contains datablocks I. DOI: 10.1107/S1600536809000725/bt2846Isup2.hkl

Additional supplementary materials:  crystallographic information; 3D view; checkCIF report

## Figures and Tables

**Table 1:** Hydrogen-bond geometry (Å, °)

*D*—H⋯*A*	*D*—H	H⋯*A*	*D*⋯*A*	*D*—H⋯*A*
O1—H1*A*⋯Cl3^i^	1.033	2.21	3.150 (3)	149.79 (16)
O1—H1*B*⋯Cl4^ii^	0.924	2.31	3.139 (3)	149.3 (2)

Symmetry codes: (i) 
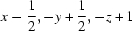
; (ii) 
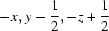
.

**Fig. 1.**
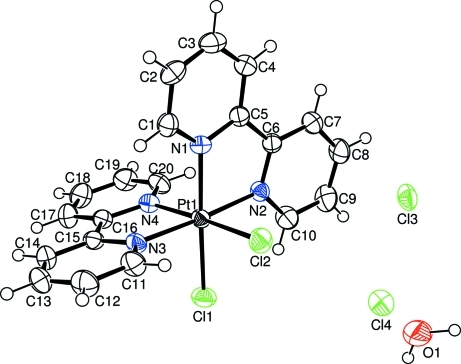
The structure of the title compound, with displacement ellipsoids drawn at the 50% probability level for non-H atoms.

## supplementary crystallographic information

### Comment

In the title complex, [PtCl_2_(C_10_H_8_N_2_)_2_]Cl_2_.H_2_O, the central Pt^4+^ ion is six-coordinated in a distorted octahedral environment by four N atoms from the two 2,2'-bipyridine ligands and two Cl atoms (Fig. 1). The main contributions to the distortion are the tight N—Pt—N chelate angles (80.33 (10)° and 80.30 (10)°), which result in non-linear *trans* axes (<Cl1—Pt1—N1 = 176.73 (7)°, <Cl2—Pt1—N4 = 176.91 (7)° and <N2—Pt1—N3 = 176.52 (10)°).

Because of the different *trans* influences of the N and Cl atoms, the Pt—N bonds *trans* to the Cl atom (lengths: 2.040 (2) and 2.037 (3) Å) are slightly longer than those *trans* to the N atom (lengths: 2.029 (2) and 2.028 (2) Å).

The compound displays intermolecular hydrogen bonding between the solvent H_2_O molecule and the Cl anions (Table 1). There is also an intermolecular π-π interaction  between the pyridine ring containing N1 and the one containg N3 at 1/2+x,1/2-y,-z, with a centroid-centroid distance of 3.962 Å and with a dihedral angle between the ring planes of 20.3°.

### Experimental

To a solution of K_2_PtCl_6_ (0.3068 g, 0.631 mmol) in H_2_O (20 ml) was added 2,2'-bipyridine (0.0971 g, 0.622 mmol) in MeOH (10 ml), and stirred for 2 h under heating. The formed precipitate was separated by filtration and washed with water and MeOH and dried under vacuum, to give a yellow powder (0.1185 g). Crystals suitable for X-ray analysis were obtained by slow evaporation from a CH_2_Cl_2_ solution.

### Refinement

H atoms were positioned geometrically and allowed to ride on their respective parent atoms [C—H = 0.93 Å and *U*_iso_(H) = 1.2*U*_eq_(C)]. The H atoms of the solvent H_2_O molecule were located from Fourier difference maps, but not refined.

### Crystal data

**Table:**

[PtCl_2_(C_10_H_8_N_2_)_2_]Cl_2_·H_2_O	*F*(000) = 1280
*M**_r_* = 667.27	*D*_x_ = 2.011 Mg m^−^^3^
Orthorhombic, *P*2_1_2_1_2_1_	Mo *K*α radiation, λ = 0.71073 Å
Hall symbol: P 2ac 2ab	Cell parameters from 958 reflections
*a* = 11.1345 (12) Å	θ = 2.4–26.4°
*b* = 11.5867 (12) Å	µ = 6.87 mm^−^^1^
*c* = 17.0873 (19) Å	*T* = 293 K
*V* = 2204.5 (4) Å^3^	Stick, colorless
*Z* = 4	0.35 × 0.20 × 0.15 mm

### Data collection

**Table:**

Bruker SMART 1000 CCD diffractometer	4462 independent reflections
Radiation source: fine-focus sealed tube	4284 reflections with *I* > 2σ(*I*)
graphite	*R*_int_ = 0.017
φ and ω scans	θ_max_ = 26.4°, θ_min_ = 2.2°
Absorption correction: multi-scan (*SADABS*; Bruker, 2000)	*h* = −12→13
*T*_min_ = 0.251, *T*_max_ = 0.357	*k* = −14→14
12649 measured reflections	*l* = −21→16

### Refinement

**Table:**

Refinement on *F*^2^	Secondary atom site location: difference Fourier map
Least-squares matrix: full	Hydrogen site location: inferred from neighbouring sites
*R*[*F*^2^ > 2σ(*F*^2^)] = 0.016	H-atom parameters constrained
*wR*(*F*^2^) = 0.038	*w* = 1/[σ^2^(*F*_o_^2^)] where *P* = (*F*_o_^2^ + 2*F*_c_^2^)/3
*S* = 0.84	(Δ/σ)_max_ = 0.003
4462 reflections	Δρ_max_ = 0.95 e Å^−^^3^
271 parameters	Δρ_min_ = −0.53 e Å^−^^3^
0 restraints	Absolute structure: Flack (1983), 1901 Friedel pairs
Primary atom site location: structure-invariant direct methods	Flack parameter: −0.006 (4)

### Special details

**Table:**

Geometry. All e.s.d.'s (except the e.s.d. in the dihedral angle between two l.s. planes) are estimated using the full covariance matrix. The cell e.s.d.'s are taken into account individually in the estimation of e.s.d.'s in distances, angles and torsion angles; correlations between e.s.d.'s in cell parameters are only used when they are defined by crystal symmetry. An approximate (isotropic) treatment of cell e.s.d.'s is used for estimating e.s.d.'s involving l.s. planes.
Refinement. Refinement of *F*^2^ against ALL reflections. The weighted *R*-factor *wR* and goodness of fit *S* are based on *F*^2^, conventional *R*-factors *R* are based on *F*, with *F* set to zero for negative *F*^2^. The threshold expression of *F*^2^ > σ(*F*^2^) is used only for calculating *R*-factors(gt) *etc*. and is not relevant to the choice of reflections for refinement. *R*-factors based on *F*^2^ are statistically about twice as large as those based on *F*, and *R*- factors based on ALL data will be even larger.

### Fractional atomic coordinates and isotropic or equivalent isotropic displacement parameters (Å^2^)

**Table:**

	*x*	*y*	*z*	*U*_iso_*/*U*_eq_	
Pt1	0.520023 (9)	0.241999 (9)	0.124231 (6)	0.02622 (4)	
Cl1	0.35373 (7)	0.12668 (7)	0.10471 (5)	0.0410 (2)	
Cl2	0.40172 (7)	0.40216 (7)	0.14570 (5)	0.0394 (2)	
Cl3	0.36627 (9)	0.07710 (8)	0.54638 (6)	0.0517 (2)	
Cl4	0.15636 (8)	0.59942 (8)	0.20502 (5)	0.0421 (2)	
N1	0.6674 (2)	0.3407 (2)	0.14792 (15)	0.0272 (6)	
N2	0.5239 (2)	0.2274 (2)	0.24255 (14)	0.0290 (5)	
N3	0.5272 (2)	0.2554 (2)	0.00599 (14)	0.0288 (5)	
N4	0.6210 (2)	0.1002 (2)	0.09926 (15)	0.0309 (6)	
C1	0.7301 (3)	0.3999 (3)	0.0943 (2)	0.0340 (7)	
H1	0.7110	0.3925	0.0416	0.041*	
C2	0.8225 (3)	0.4716 (3)	0.1167 (2)	0.0389 (8)	
H2	0.8644	0.5140	0.0794	0.047*	
C3	0.8524 (3)	0.4801 (3)	0.1945 (2)	0.0413 (8)	
H3	0.9159	0.5270	0.2099	0.050*	
C4	0.7876 (3)	0.4185 (3)	0.2500 (2)	0.0364 (8)	
H4	0.8078	0.4227	0.3027	0.044*	
C5	0.6933 (3)	0.3516 (3)	0.22561 (18)	0.0284 (7)	
C6	0.6124 (3)	0.2891 (3)	0.27847 (18)	0.0282 (7)	
C7	0.6188 (3)	0.2940 (3)	0.35849 (19)	0.0391 (8)	
H7	0.6792	0.3364	0.3827	0.047*	
C8	0.5347 (3)	0.2355 (3)	0.4032 (2)	0.0415 (8)	
H8	0.5378	0.2380	0.4575	0.050*	
C9	0.4458 (3)	0.1728 (3)	0.3651 (2)	0.0452 (9)	
H9	0.3886	0.1327	0.3940	0.054*	
C10	0.4424 (3)	0.1702 (3)	0.2852 (2)	0.0399 (8)	
H10	0.3827	0.1281	0.2601	0.048*	
C11	0.4779 (3)	0.3413 (3)	−0.0360 (2)	0.0386 (8)	
H11	0.4407	0.4024	−0.0103	0.046*	
C12	0.4818 (3)	0.3402 (3)	−0.1165 (2)	0.0453 (9)	
H12	0.4465	0.3996	−0.1450	0.054*	
C13	0.5380 (3)	0.2508 (3)	−0.15455 (19)	0.0413 (8)	
H13	0.5409	0.2489	−0.2089	0.050*	
C14	0.5907 (3)	0.1629 (3)	−0.11082 (19)	0.0371 (8)	
H14	0.6302	0.1024	−0.1356	0.045*	
C15	0.5836 (3)	0.1666 (3)	−0.03080 (19)	0.0294 (7)	
C16	0.6334 (3)	0.0775 (3)	0.02149 (18)	0.0299 (7)	
C17	0.6884 (3)	−0.0219 (3)	−0.0035 (2)	0.0404 (8)	
H17	0.6942	−0.0385	−0.0566	0.048*	
C18	0.7351 (3)	−0.0971 (3)	0.0517 (2)	0.0444 (9)	
H18	0.7734	−0.1644	0.0357	0.053*	
C19	0.7249 (3)	−0.0725 (3)	0.1296 (2)	0.0435 (8)	
H19	0.7567	−0.1223	0.1669	0.052*	
C20	0.6665 (3)	0.0280 (3)	0.1524 (2)	0.0366 (8)	
H20	0.6589	0.0451	0.2054	0.044*	
O1	0.0454 (3)	0.2475 (2)	0.37482 (18)	0.0807 (11)	
H1A	0.0141	0.3103	0.4125	0.080*	
H1B	−0.0004	0.1831	0.3636	0.080*	

### Atomic displacement parameters (Å^2^)

**Table:**

	*U*^11^	*U*^22^	*U*^33^	*U*^12^	*U*^13^	*U*^23^
Pt1	0.02736 (6)	0.02822 (6)	0.02310 (6)	−0.00141 (5)	−0.00187 (4)	0.00024 (5)
Cl1	0.0367 (4)	0.0447 (5)	0.0416 (5)	−0.0117 (4)	−0.0046 (3)	−0.0014 (4)
Cl2	0.0400 (4)	0.0375 (4)	0.0408 (5)	0.0082 (4)	−0.0011 (3)	−0.0036 (3)
Cl3	0.0656 (6)	0.0536 (6)	0.0360 (5)	−0.0087 (5)	0.0145 (4)	−0.0081 (4)
Cl4	0.0430 (5)	0.0454 (5)	0.0379 (5)	0.0001 (4)	−0.0017 (4)	−0.0040 (4)
N1	0.0243 (13)	0.0257 (13)	0.0314 (16)	−0.0014 (10)	−0.0031 (11)	0.0011 (10)
N2	0.0319 (13)	0.0301 (13)	0.0251 (13)	−0.0025 (14)	0.0013 (10)	0.0010 (10)
N3	0.0294 (13)	0.0332 (13)	0.0238 (12)	0.0022 (16)	−0.0056 (9)	0.0013 (10)
N4	0.0294 (14)	0.0309 (14)	0.0325 (15)	−0.0022 (11)	−0.0021 (11)	−0.0006 (11)
C1	0.0361 (18)	0.0362 (18)	0.0297 (18)	0.0021 (15)	0.0030 (14)	0.0024 (14)
C2	0.0323 (17)	0.0387 (18)	0.046 (2)	−0.0046 (13)	0.0074 (17)	0.0082 (17)
C3	0.0320 (18)	0.0377 (19)	0.054 (2)	−0.0076 (15)	−0.0029 (16)	−0.0009 (16)
C4	0.0338 (18)	0.042 (2)	0.033 (2)	−0.0010 (15)	−0.0042 (14)	−0.0032 (15)
C5	0.0288 (16)	0.0294 (16)	0.0271 (17)	0.0042 (13)	−0.0025 (13)	0.0009 (13)
C6	0.0277 (16)	0.0301 (15)	0.0269 (16)	0.0028 (12)	−0.0013 (12)	−0.0002 (12)
C7	0.0398 (19)	0.0493 (19)	0.0281 (19)	−0.0004 (15)	−0.0020 (14)	−0.0022 (15)
C8	0.050 (2)	0.050 (2)	0.0240 (16)	0.003 (2)	0.0016 (13)	0.0056 (14)
C9	0.058 (2)	0.0409 (19)	0.037 (2)	−0.0088 (16)	0.0134 (18)	0.0074 (16)
C10	0.047 (2)	0.0370 (18)	0.035 (2)	−0.0099 (16)	0.0033 (16)	0.0032 (14)
C11	0.044 (2)	0.0380 (17)	0.0336 (19)	0.0054 (17)	−0.0062 (16)	0.0047 (14)
C12	0.051 (2)	0.0494 (19)	0.035 (2)	0.0019 (17)	−0.0079 (18)	0.0104 (16)
C13	0.0481 (19)	0.052 (2)	0.0233 (15)	0.000 (3)	−0.0028 (13)	0.0036 (15)
C14	0.0406 (19)	0.0441 (18)	0.0267 (19)	−0.0010 (15)	0.0029 (15)	−0.0038 (14)
C15	0.0268 (16)	0.0338 (17)	0.0277 (17)	−0.0020 (13)	−0.0035 (13)	0.0011 (13)
C16	0.0288 (17)	0.0327 (17)	0.0283 (18)	−0.0023 (13)	−0.0017 (13)	−0.0008 (13)
C17	0.045 (2)	0.039 (2)	0.037 (2)	0.0053 (17)	0.0047 (15)	−0.0058 (15)
C18	0.044 (2)	0.038 (2)	0.051 (3)	0.0120 (17)	0.0043 (17)	−0.0011 (17)
C19	0.0421 (19)	0.0395 (18)	0.049 (2)	0.0079 (15)	−0.0068 (18)	0.0024 (19)
C20	0.0423 (19)	0.0386 (18)	0.0287 (18)	−0.0012 (15)	−0.0087 (15)	0.0029 (14)
O1	0.0528 (17)	0.084 (2)	0.106 (3)	−0.0042 (15)	−0.0045 (15)	−0.043 (2)

### Geometric parameters (Å, °)

**Table:**

Pt1—N3	2.028 (2)	C7—H7	0.9300
Pt1—N2	2.029 (2)	C8—C9	1.389 (5)
Pt1—N4	2.037 (3)	C8—H8	0.9300
Pt1—N1	2.040 (2)	C9—C10	1.366 (5)
Pt1—Cl2	2.3051 (8)	C9—H9	0.9300
Pt1—Cl1	2.3076 (8)	C10—H10	0.9300
N1—C1	1.341 (4)	C11—C12	1.377 (5)
N1—C5	1.364 (4)	C11—H11	0.9300
N2—C10	1.339 (4)	C12—C13	1.375 (5)
N2—C6	1.364 (4)	C12—H12	0.9300
N3—C11	1.344 (4)	C13—C14	1.393 (5)
N3—C15	1.360 (4)	C13—H13	0.9300
N4—C20	1.335 (4)	C14—C15	1.370 (4)
N4—C16	1.361 (4)	C14—H14	0.9300
C1—C2	1.377 (4)	C15—C16	1.474 (4)
C1—H1	0.9300	C16—C17	1.373 (4)
C2—C3	1.373 (5)	C17—C18	1.385 (5)
C2—H2	0.9300	C17—H17	0.9300
C3—C4	1.389 (5)	C18—C19	1.367 (5)
C3—H3	0.9300	C18—H18	0.9300
C4—C5	1.370 (4)	C19—C20	1.389 (4)
C4—H4	0.9300	C19—H19	0.9300
C5—C6	1.467 (4)	C20—H20	0.9300
C6—C7	1.370 (4)	O1—H1A	1.033
C7—C8	1.385 (5)	O1—H1B	0.924
			
N3—Pt1—N2	176.52 (10)	C7—C6—C5	124.2 (3)
N3—Pt1—N4	80.30 (10)	C6—C7—C8	119.6 (3)
N2—Pt1—N4	97.47 (10)	C6—C7—H7	120.2
N3—Pt1—N1	97.07 (10)	C8—C7—H7	120.2
N2—Pt1—N1	80.33 (10)	C7—C8—C9	118.7 (3)
N4—Pt1—N1	92.84 (9)	C7—C8—H8	120.7
N3—Pt1—Cl2	96.85 (7)	C9—C8—H8	120.7
N2—Pt1—Cl2	85.44 (7)	C10—C9—C8	119.9 (3)
N4—Pt1—Cl2	176.91 (7)	C10—C9—H9	120.1
N1—Pt1—Cl2	88.68 (7)	C8—C9—H9	120.1
N3—Pt1—Cl1	86.10 (7)	N2—C10—C9	121.0 (3)
N2—Pt1—Cl1	96.47 (7)	N2—C10—H10	119.5
N4—Pt1—Cl1	86.88 (7)	C9—C10—H10	119.5
N1—Pt1—Cl1	176.73 (7)	N3—C11—C12	120.9 (3)
Cl2—Pt1—Cl1	91.76 (3)	N3—C11—H11	119.5
C1—N1—C5	120.5 (3)	C12—C11—H11	119.5
C1—N1—Pt1	124.8 (2)	C13—C12—C11	119.6 (3)
C5—N1—Pt1	114.53 (19)	C13—C12—H12	120.2
C10—N2—C6	120.3 (3)	C11—C12—H12	120.2
C10—N2—Pt1	124.7 (2)	C12—C13—C14	119.3 (3)
C6—N2—Pt1	114.83 (19)	C12—C13—H13	120.3
C11—N3—C15	120.2 (3)	C14—C13—H13	120.3
C11—N3—Pt1	124.9 (2)	C15—C14—C13	119.2 (3)
C15—N3—Pt1	114.88 (19)	C15—C14—H14	120.4
C20—N4—C16	120.3 (3)	C13—C14—H14	120.4
C20—N4—Pt1	124.9 (2)	N3—C15—C14	120.8 (3)
C16—N4—Pt1	114.6 (2)	N3—C15—C16	115.1 (3)
N1—C1—C2	120.6 (3)	C14—C15—C16	124.1 (3)
N1—C1—H1	119.7	N4—C16—C17	120.7 (3)
C2—C1—H1	119.7	N4—C16—C15	114.8 (3)
C3—C2—C1	119.5 (3)	C17—C16—C15	124.5 (3)
C3—C2—H2	120.2	C16—C17—C18	118.9 (3)
C1—C2—H2	120.2	C16—C17—H17	120.6
C2—C3—C4	119.9 (3)	C18—C17—H17	120.6
C2—C3—H3	120.0	C19—C18—C17	120.1 (3)
C4—C3—H3	120.0	C19—C18—H18	119.9
C5—C4—C3	118.7 (3)	C17—C18—H18	119.9
C5—C4—H4	120.6	C18—C19—C20	119.1 (3)
C3—C4—H4	120.6	C18—C19—H19	120.4
N1—C5—C4	120.7 (3)	C20—C19—H19	120.4
N1—C5—C6	115.0 (3)	N4—C20—C19	120.8 (3)
C4—C5—C6	124.2 (3)	N4—C20—H20	119.6
N2—C6—C7	120.6 (3)	C19—C20—H20	119.6
N2—C6—C5	115.2 (3)	H1A—O1—H1B	120.8
			
N3—Pt1—N1—C1	6.2 (3)	C3—C4—C5—C6	−175.4 (3)
N2—Pt1—N1—C1	−176.1 (3)	C10—N2—C6—C7	−0.4 (4)
N4—Pt1—N1—C1	86.8 (3)	Pt1—N2—C6—C7	174.8 (2)
Cl2—Pt1—N1—C1	−90.5 (2)	C10—N2—C6—C5	−177.7 (3)
N3—Pt1—N1—C5	−179.5 (2)	Pt1—N2—C6—C5	−2.6 (3)
N2—Pt1—N1—C5	−1.9 (2)	N1—C5—C6—N2	1.0 (4)
N4—Pt1—N1—C5	−99.0 (2)	C4—C5—C6—N2	179.9 (3)
Cl2—Pt1—N1—C5	83.7 (2)	N1—C5—C6—C7	−176.2 (3)
N4—Pt1—N2—C10	−91.0 (3)	C4—C5—C6—C7	2.7 (5)
N1—Pt1—N2—C10	177.4 (3)	N2—C6—C7—C8	0.2 (5)
Cl2—Pt1—N2—C10	87.9 (2)	C5—C6—C7—C8	177.3 (3)
Cl1—Pt1—N2—C10	−3.3 (3)	C6—C7—C8—C9	0.1 (5)
N4—Pt1—N2—C6	94.0 (2)	C7—C8—C9—C10	−0.2 (5)
N1—Pt1—N2—C6	2.4 (2)	C6—N2—C10—C9	0.3 (5)
Cl2—Pt1—N2—C6	−87.0 (2)	Pt1—N2—C10—C9	−174.4 (3)
Cl1—Pt1—N2—C6	−178.28 (19)	C8—C9—C10—N2	0.0 (5)
N4—Pt1—N3—C11	−178.1 (3)	C15—N3—C11—C12	1.1 (5)
N1—Pt1—N3—C11	−86.4 (3)	Pt1—N3—C11—C12	−176.5 (2)
Cl2—Pt1—N3—C11	3.1 (2)	N3—C11—C12—C13	−0.8 (5)
Cl1—Pt1—N3—C11	94.4 (2)	C11—C12—C13—C14	−0.3 (5)
N4—Pt1—N3—C15	4.2 (2)	C12—C13—C14—C15	1.0 (5)
N1—Pt1—N3—C15	95.9 (2)	C11—N3—C15—C14	−0.3 (4)
Cl2—Pt1—N3—C15	−174.61 (19)	Pt1—N3—C15—C14	177.5 (2)
Cl1—Pt1—N3—C15	−83.3 (2)	C11—N3—C15—C16	−179.7 (3)
N3—Pt1—N4—C20	178.5 (3)	Pt1—N3—C15—C16	−1.8 (3)
N2—Pt1—N4—C20	1.2 (3)	C13—C14—C15—N3	−0.7 (5)
N1—Pt1—N4—C20	81.8 (3)	C13—C14—C15—C16	178.6 (3)
Cl1—Pt1—N4—C20	−94.9 (3)	C20—N4—C16—C17	2.6 (5)
N3—Pt1—N4—C16	−6.0 (2)	Pt1—N4—C16—C17	−173.2 (2)
N2—Pt1—N4—C16	176.7 (2)	C20—N4—C16—C15	−177.5 (3)
N1—Pt1—N4—C16	−102.7 (2)	Pt1—N4—C16—C15	6.8 (3)
Cl1—Pt1—N4—C16	80.5 (2)	N3—C15—C16—N4	−3.3 (4)
C5—N1—C1—C2	0.8 (5)	C14—C15—C16—N4	177.4 (3)
Pt1—N1—C1—C2	174.7 (2)	N3—C15—C16—C17	176.6 (3)
N1—C1—C2—C3	1.6 (5)	C14—C15—C16—C17	−2.7 (5)
C1—C2—C3—C4	−1.5 (5)	N4—C16—C17—C18	−2.3 (5)
C2—C3—C4—C5	−1.0 (5)	C15—C16—C17—C18	177.8 (3)
C1—N1—C5—C4	−3.4 (4)	C16—C17—C18—C19	0.7 (5)
Pt1—N1—C5—C4	−177.9 (2)	C17—C18—C19—C20	0.6 (5)
C1—N1—C5—C6	175.6 (3)	C16—N4—C20—C19	−1.3 (5)
Pt1—N1—C5—C6	1.1 (3)	Pt1—N4—C20—C19	174.0 (2)
C3—C4—C5—N1	3.4 (5)	C18—C19—C20—N4	−0.3 (5)

### Hydrogen-bond geometry (Å, °)

**Table:**

*D*—H···*A*	*D*—H	H···*A*	*D*···*A*	*D*—H···*A*
O1—H1A···Cl3^i^	1.033	2.21	3.150 (3)	149.79 (16)
O1—H1B···Cl4^ii^	0.924	2.31	3.139 (3)	149.3 (2)

Symmetry codes: (i) *x*−1/2, −*y*+1/2, −*z*+1; (ii) −*x*, *y*−1/2, −*z*+1/2.
